# Impact of text reminders on pneumatic compression device (PCD) compliance in patients with breast cancer-related lymphedema

**DOI:** 10.1007/s00520-023-08246-9

**Published:** 2023-12-16

**Authors:** Shail Maingi, Ellen M. O’Malley

**Affiliations:** 1 St Peter’s Health Partners, Albany, NY 12208 USA; 2 Dana-Farber Cancer Institute, 101 Columbian St., South Weymouth, MA 02190 USA; 3 O’Malley Medical Communications, Victoria, MN 55386 USA

**Keywords:** Breast cancer-related lymphedema, Pneumatic compression device, Quality of life measures, Treatment compliance

## Abstract

**Purpose:**

Do cell phone text reminders impact the rate of compliance with pneumatic compression device (PCD) therapy among women with breast cancer-related lymphedema (BCRL)?

**Methods:**

A prospective, randomized, 2-group feasibility study conducted at 2 centers. Participants were adult females (≥18 years old) with unilateral BCRL who had the capability of receiving reminder text messages. All participants underwent PCD therapy. Participants were randomized 1:1 to control (no text messages) or test group (received text message reminders if the PCD had not been used for 2 consecutive days). The rate of compliance between treatment groups was the main outcome measure. Secondary outcome measures were changes in arm girth, quality of life (QOL), and symptom severity.

**Results:**

Twenty-nine participants were enrolled and randomized, 25 were available for follow-up at 60 days (14 test, 11 control). Overall, 52.2% (12/23) of all participants were completely compliant, an additional 43.5% (10/23) were partially compliant, and 1 patient (4.3%) was noncompliant. The test and control groups did not differ in device compliance. In the pooled population, weight, BMI, and arm girth were improved. Overall disease-specific QOL and symptom severity were improved. Regression analysis showed benefits were greater among participants with higher rates of compliance.

**Conclusions:**

Automated text reminders did not improve compliance in patients with BCRL as compliance rates were already high in this patient population. Improvements in weight, BMI, arm girth, disease-specific quality of life, and symptom severity measures were observed regardless of the treatment assignment. Full compliance resulted in greater functional and QOL benefits.

**Trial registration:**

The study was registered at www.clinicaltrials.gov (NCT04432727) on June 16, 2020.

## Introduction

Lymphedema is a chronic debilitating disease marked by deficits in lymph drainage and accumulation of protein-rich fluid, leading to limb edema that can progress to cellulitis and fibrosis over time. People with lymphedema are susceptible to extremity impairment, recurrent soft tissue inflammation and infections, lymphorrhea, body disfigurement, and psychological and social issues [1, 2]. Breast cancer treatments can result in breast cancer-related lymphedema (BCRL), with reported frequencies ranging from 6 to 65% [3–5].

Treatments for lymphedema focus on symptom management and improved patient-reported outcomes (PROs). Traditional interventions include manual lymphatic drainage (MLD), compression therapy and self-care (e.g., skin hygiene, limb elevation, exercise, compression garments) [6]. In very severe cases, lymphatic exchange may be performed [7]. More recently, pneumatic compression devices (PCDs) have become an additional treatment option that clinicians can offer patients for the treatment of lymphedema. Clinical studies have demonstrated that regular use of PCDs, as an adjunct to standard self-care measures, is associated with significant patient-reported improvements in overall symptoms, decreased limb girth, decreased limb volume, increased elasticity of tissues, and fewer episodes of infection [8–12].

Adherence to prescribed at-home self-care is critical to the successful treatment of lymphedema [11, 13]. Research shows that compliance to some risk management behaviors diminishes over time while adherence to other behaviors remains high [14, 15]. Text messaging is a convenient method to send patients reminders to use home therapies, take prescribed medications, or follow postoperative instructions and can been used for a variety of disease states [16–18]. The primary purpose of this study was to determine whether cell phone text reminders impacted the rate of compliance with PCD therapy. Secondary outcomes were to examine the changes in arm girth, quality of life, and symptom severity in patients using PCD for BCRL.

## Materials and methods

### Study design and population

The study was an on-label, prospective, randomized, 2-group feasibility study conducted at 2 centers. Ethics approval was received through Western IRB (Puyallup, WA) and University of Louisville Human Subjects Protection Program Office (Louisville, KY). The study is registered at www.clinicaltrials.gov (unique identifier: NCT04432727). Before participating in the study, all participants signed the IRB-approved written informed consent.

Participants were females of 18 years or older with unilateral BCRL who provided informed consent, agreed to comply with the study requirements, and were able to receive text messages from the study sponsor. Participants were excluded from participation if they had used a PCD in the previous 3 months, had undergone phase 1 complete decompression therapy (CDT) within 1 month or were planning to undergo during the study period, were currently undergoing curative cancer therapy, or were unable to be fitted for PCD garments. Additional medical conditions excluding participants were heart failure, acute venous disease, active skin or limb infection or inflammatory disease, pregnancy or planning to become pregnant, and any condition where increased lymphatic or venous return was undesirable. Lastly, participants were excluded if there was a known inability to receive cell phone connection where the PCD therapy was to be administered.

After signing the informed consent and inclusion/exclusion criteria were confirmed, participants were randomized by an electronic data capture database to either PCD therapy with connectivity (test) or PCD therapy without connectivity (control). The randomization scheme was generated by a statistician using a permutated block design with block size balanced within each block to maintain a 1:1 ratio between treatment groups.

PCD therapy was conducted using the Flexitouch® (FT) Plus advanced PCD (Tactile Medical, Minneapolis, MN, USA) in daily 60-min U1 unilateral sessions at normal pressure. The devices used for the study were identical to the commercially available devices except for a cellular communication module that was added to the controller unit, which transmitted usage data to a cloud-based database. This usage information was used to send automated text message reminders to participants in the test group if they had not used the device for 2 consecutive days. Participants in the control group did not receive text message reminders.

### Assessments

Participants completed study visits at screening, baseline, device training (within 21 days after the baseline visit), and at 30-day and 60-day follow-ups. Device training was conducted by qualified Tactile Medical personnel.

The primary endpoint was to compare the rate of treatment compliance in patients in the test group with those in the control group. Complete compliance was defined as an average of 5–7 treatments per week, partial compliance as 1–4 treatments per week, and noncompliance as <1 treatment per week.

Exploratory endpoints included assessments of arm girth, quality of life (QOL), symptom questionnaires, and adverse events. Participants were provided with a tape measure to make arm girth measurements on the anterior forearm 6 cm below the midline of the antecubital fossa on the affected arm.

Disease-specific quality of life was measured using the Lymphedema Quality of Life Tool (LYMQOL ARM). The LYMQOL ARM is a 21-item questionnaire designed and validated in patients with chronic edema. The survey includes 4 domains: function, appearance, symptoms, and mood, and an overall QOL score. Each domain item is scored on a 4-point scale with higher scores indicating worse QOL. The overall QOL score is scored from 0 (poor) to 10 (excellent) [19].

Symptom severity was assessed using the Lymphedema Symptom Intensity and Distress Survey-Arm (LSIDS-A). The LSIDS-A is a 30-item validated assessment tool designed for measuring arm lymphedema and its associated symptoms in patients with BCRL [20]. The questionnaire reporting period is the previous 7 days and symptoms are reported as “yes” or “no,” symptoms with responses of “yes” are then scored from 1 to 5 with higher scores indicating more severe symptoms. The scores are calculated into an overall score and 7 domain scores: soft tissue sensation, neurological sensation, functional, behavioral, resource, sexual function, and activity. The overall and domain scores are the means of the individual items included in the score.

General quality of life was assessed using the validated RAND Short Form-36 (SF-36). The SF-36 evaluates 8 domains: general health, physical functioning, physical role limitations, emotional role limitations, energy/fatigue, emotional well-being, social functioning, and bodily pain [21]. Scores were normalized, so that each score has a range from 0 (maximum disability) to 100 (no disability). Higher scores indicate a more favorable health state.

Adverse events were categorized as serious or nonserious with severity rankings of mild, moderate, or severe. The relationship of the event to the device was rated as not related, possibly, probably, or definitely related.

### Statistical analysis

No power calculations were used to derive a sample size given this study sought to identify which health outcomes should be used for a larger randomized controlled trial in the future. The desired target sample size for this feasibility study was set at 10 analyzable data sets for each group. Enrollment of up to 30 participants was planned to achieve 60-day follow-up data on at least 20 participants.

The analysis population includes all enrolled participants. Participants were assessed by treatment group for the primary endpoint. Ad hoc regression analyses were performed on the pooled cohort by compliance status (complete, partial, noncompliance), irrespective of the treatment assignment.

Descriptive statistics were calculated for all continuous variables. Frequencies, percentages, and confidence intervals were calculated for categorical data. Any data found to be randomly missing within the survey measures were handled as specified by the survey developers. Nonrandom missing visit data were not imputed.

Mixed effects regression models were fit to the data to account for the repeated (non-independent) nature of the measurements for each participant over multiple timepoints. An autocorrelation structure (AR1) was used to account for single-order correlation between timepoints. For each participant, the measures post baseline are likely dependent upon the prior timepoint. The post baseline means and differences presented in the tables are model estimated means and differences, and not the raw observed means and differences for each time point. A Tukey method was used to adjust the confidence intervals and *p* values for multiple comparisons. *P* values <0.05 were considered statistically significant.

Analyses were performed using R version 4.1.0 or higher (The R Foundation for Statistical Computing. https://www.R-Project.org).

## Results

Sixty-one participants were screened for the study with 29 ultimately enrolled and randomized between August 2020 and September 2021. Four participants withdrew, resulting in 60-day follow-up available for 25 participants (14 test, 11 control).

Demographic and other baseline data are presented Table 1. Participants were primarily White women (82.8%) in their 50’s with BMI >30. The test group had a shorter period since their lymphedema diagnosis than the control group (1.7 vs 2.5 years).

**Table 1:** Demographics and baseline characteristics by group

Characteristic		Test *N*=15	Control *N*=14	All Participants *N*=29
Age (years)		52.3 (10.8)	57.7 (9.7)	54.9 (10.4)
Body mass index (kg/m^2^)		33.1 (8.0)	32.4 (7.4)	32.8 (7.6)
Ethnicity	Hispanic/Latino	0.0% (0)	14.3% (2)	6.9% (2)
	Non-Hispanic/Latino	100% (15)	78.6% (11)	89.7% (26)
	Unknown	0.0% (0)	7.1% (1)	3.4% (1)
Race	Black	13.3% (2)	21.4% (3)	17.2% (5)
	White or Caucasian	86.7% (13)	78.6% (11)	82.8% (24)
Time since lymphedema diagnosis (years)		1.7 (1.8)	2.5 (2.0)	2.1 (1.9)
Time since most recent surgery (years)		2.8 (2.9)	4.1 (2.7)	3.4 (2.8)

Results are presented as mean (SD) or % (n)

### Compliance with treatment

The primary endpoint of treatment compliance is shown in Table 2. Two participants, 1 in each group, did not start treatment but otherwise continued in the study; hence, only 23 participants have device compliance data. One test group participant did not receive an expected text reminder due to technical issues. There was no difference between the test and control groups for device compliance. In addition, no statistically significant differences in compliance were seen based on demographic or baseline characteristics. Overall, 52.2% (12/23) of participants were completely compliant, an additional 43.5% (10/23) were partially compliant, and 1 patient (4.3%) was noncompliant. Since there was no difference in compliance between the treatment groups, outcomes were analyzed on the complete cohort.

**Table 2:** Treatment compliance at 60 days by group

Compliance		Test *N*=13	Control *N*=10	All Participants^a^ *N*=23
Treatments per week		5.0 ± 2.0	4.8 ± 2.3	4.9 ± 2.1
Noncompliant	(<1 day/week)	0.0% (0)	10.0% (1)	4.3% (1)
Partially compliant	1-4 days /week	46.2% (6)	40.0% (4)	43.5% (10)
Completely compliant	5-7 days/week	53.8% (7)	50.0% (5)	52.2% (12)

Results are presented as mean ± SD or % (*n*)

^a^Two participants, 1 in each group, did not start PCD treatment, and therefore, have no compliance data

### Outcomes for pooled population

Changes in weight, BMI, and arm girth are shown in Table 3. By regression analysis, the reductions in weight and BMI at the 30-day visit were statistically significant (*p*<0.05). The change in arm girth was not significant at the 30-day visit, but was significantly reduced at the 60-day visit (*p*=0.034).

**Table 3:** Changes in weight, BMI, and arm girth in pooled population

Measure/Time Period	Model Estimate [95% CI]	Change from Baseline	*P* value
Weight (lb)			
Baseline	185.2 [169.3, 201.1]		
30-Day	182.9 [167.0, 198.8]	–2.3	**0.011**
60-Day	182.9 [167.0, 198.8]	–2.3	0.066
BMI (kg/m^2^)			
Baseline	32.8 [29.9, 35.6]		
30-Day	32.3 [29.5, 35.2]	–0.4	**0.011**
60-Day	32.4 [29.5, 35.2]	–0.4	0.069
Arm Girth (cm)			
Baseline	28.0 [26.6, 29.4]		
30-Day	27.4 [25.9, 28.8]	–0.6	0.295
60-Day	27.0 [25.5, 28.4]	–1.0	**0.034**

A mixed effects regression model was fit to the data to account for the repeated (non-independent) nature of the measurements for each participant over multiple timepoints. Random intercepts (but not random slopes) were included in the model specification. An autocorrelation structure (AR1) was used to account for single order correlations between timepoints. For each participant, the measures post baseline are likely dependent upon the preceding timepoint, while also allowing for the magnitude of the correlation to decline over time. A Tukey method was used to adjust the confidence intervals and *p* values for multiple comparisons. The means and differences presented in the table are model estimated means and differences, and not the raw observed means and differences for each timepoint. Bold *p* values indicate statistically significant changes from baseline

The overall and domain scores of the LYMQOL ARM questionnaire are provided in Table 4. The overall quality of life and functional domain scores were significantly improved at the 60-day follow-up (*p*=0.004 and *p*=0.027, respectively). The symptom domain score was significantly improved at 30-day follow-up (*p*=0.006). The mood domain was also significantly improved at the 30-day follow-up (*p*=0.009).

**Table 4:** LYMQOL ARM scores in pooled population

Domain/Time Period	Model Estimate [95% CI]	Change from Baseline	*P* value
Overall Quality of Life			
Baseline	6.5 [5.7, 7.2]		
30-Day	6.8 [6.0, 7.6]	0.33	0.432
60-Day	7.3 [6.5, 8.1]	0.86	**0.004**
Function Domain			
Baseline	1.8 [1.6, 2.0]		
30-Day	1.6 [1.4, 1.8]	–0.18	0.073
60-Day	1.5 [1.3, 1.7]	–0.28	**0.027**
Appearance Domain			
Baseline	1.9 [1.7, 2.2]		
30-Day	1.8 [1.5, 2.0]	–0.20	0.060
60-Day	1.7 [1.4, 2.0]	–0.27	0.075
Symptoms Domain			
Baseline	2.4 [2.2, 2.7]		
30-Day	2.2 [1.9, 2.4]	–0.30	**0.006**
60-Day	2.2 [1.9, 2.4]	–0.30	0.054
Mood Domain			
Baseline	2.0 [1.7, 2.2]		
30-Day	1.7 [1.4, 1.9]	–0.29	**0.009**
60-Day	1.7 [1.4, 2.0]	–0.23	0.108

The overall score is a single question with response ranging from 0 (poor) to 10 (excellent). The other items are scored on a 4-point scale with higher scores indicating worse QOL. Each domain score is the mean score of the items included in that domain

A mixed effects regression model was fit to the data to account for the repeated (non-independent) nature of the measurements for each participant over multiple timepoints. Random intercepts (but not random slopes) were included in the model specification. An autocorrelation structure (AR1) was used to account for single order correlations between timepoints. For each participant, the measures post baseline are likely dependent upon the preceding timepoint, while also allowing for the magnitude of the correlation to decline over time. A Tukey method was used to adjust the confidence intervals and *p* values for multiple comparisons. The means and differences presented in the table are model estimated means and differences, and not the raw observed means and differences for each timepoint. Bold *p* values indicate statistically significant changes from baseline

The LSIDS-A results (Table 5) indicate that overall score improved significantly at both timepoints, as did the neurological sensation domain score. The soft tissue sensation domain and behavioral domain scores were significantly improved at the 60-day follow-up (*p*=0.010, and *p*=0.044, respectively).

**Table 5:** LSIDS-A scores in pooled population

Domain/Time Period	Model Estimate [95% CI]	Mean Change from Baseline	*P* value
Overall Domain			
Baseline	2.8 [2.1, 3.5]		
30-Day	2.2 [1.5, 3.0]	–0.57	**0.018**
60-Day	1.9 [1.2, 2.7]	–0.88	**0.001**
Soft Tissue Sensation Domain			
Baseline	3.9 [3.1, 4.6]		
30-Day	3.0 [2.2, 3.8]	–0.85	0.069
60-Day	2.4 [1.6, 3.2]	–1.43	**0.010**
Neurological Sensation Domain			
Baseline	3.4 [2.5, 4.2]		
30-Day	2.3 [1.5, 3.2]	–1.1	**0.016**
60-Day	1.9 [1.0, 2.8]	–1.4	**0.004**
Functional Domain			
Baseline	2.1 [1.2, 3.1]		
30-Day	1.7 [0.8, 2.7]	–0.38	0.480
60-Day	1.5 [0.6, 2.5]	–0.60	0.135
Behavioral Domain			
Baseline	2.7 [1.7, 3.6]		
30-Day	2.2 [1.2, 3.1]	–0.48	0.089
60-Day	2.1 [1.1, 3.0]	–0.58	**0.044**
Resource Domain			
Baseline	1.3 [0.3, 2.3]		
30-Day	1.0 [0.0, 2.0]	–0.34	0.624
60-Day	0.9 [–0.1, 1.9]	–0.41	0.391
Sexual Function Domain			
Baseline	2.0 [0.9, 3.2]		
30-Day	1.9 [0.8, 3.1]	–0.10	0.976
60-Day	1.5 [0.3, 2.7]	–0.56	0.568
Activity Domain			
Baseline	2.9 [1.8, 4.1]		
30-Day	2.8 [1.6, 4.0]	–0.14	0.913
60-Day	2.3 [1.2, 3.5]	–0.59	0.127

Symptoms are indicated by yes/no answers; responses of “yes” are scored from 1 to 5 with higher scores indicating more severe symptoms

A mixed effects regression model was fit to the data to account for the repeated (non-independent) nature of the measurements for each participant over multiple timepoints. Random intercepts (but not random slopes) were included in the model specification. An autocorrelation structure (AR1) was used to account for single-order correlations between timepoints. For each participant, the measures post baseline are likely dependent upon the preceding timepoint, while also allowing for the magnitude of the correlation to decline over time. A Tukey method was used to adjust the confidence intervals and *p* values for multiple comparisons. The means and differences presented in the table are model estimated means and differences, and not the raw observed means and differences for each timepoint. Bold *p* values indicate statistically significant changes from baseline

The SF-36 scores results are presented in Table 6. Although improvements from baseline were observed in each domain, only the change in the pain domain was statistically significant at the 30-day (*p*=0.042) and 60-day (*p*=0.009) follow-ups.

**Table 6:** RAND SF-36 scores in pooled population

Domain/Time Period	Model Estimate [95% CI]	Mean Change from Baseline	*P* value
General Health Domain			
Baseline	56.4 [46.5, 66.3]		
30-Day	61.7 [51.7, 71.7]	5.3	0.158
60-Day	61.7 [51.6, 71.9]	5.3	0.253
Physical Functioning Domain			
Baseline	56.4 [46.5, 66.3]		
30-Day	61.7 [51.7, 71.7]	5.3	0.158
60-Day	61.7 [51.6, 71.9]	5.3	0.253
Physical Role Limitations Domain			
Baseline	47.4 [31.0, 63.8]		
30-Day	45.6 [28.8, 62.5]	–1.8	0.979
60-Day	63.6 [46.5, 80.8]	16.2	0.098
Emotional Role Limitations Domain			
Baseline	61.4 [48.5, 80.3]		
30-Day	64.8 [48.4, 81.1]	0.4	1.000
60-Day	69.9 [53.2, 86.7]	5.6	0.770
Energy/Fatigue Domain			
Baseline	47.1 [39.8, 54.3]		
30-Day	48.2 [40.8, 55.6]	1.2	0.934
60-Day	48.3 [40.7, 55.9]	1.2	0.935
Emotional Well-being Domain			
Baseline	68.7 [61.9, 75.5]		
30-Day	69.0 [62.1, 75.9]	0.3	0.992
60-Day	72.1 [65.1, 79.1]	3.4	0.285
Social Functioning Domain			
Baseline	70.3 [60.5, 80.0]		
30-Day	76.9 [67.0, 86.9]	6.7	0.244
60-Day	72.7 [62.5, 82.8]	2.4	0.838
Pain Domain			
Baseline	56.2 [46.5, 65.9]		
30-Day	64.7 [54.8, 74.5]	8.5	**0.042**
60-Day	68.2 [58.1, 78.2]	12.0	**0.009**

Each domain has a scale of 0 (maximum disability) to 100 (no disability), with higher scores indicating a more favorable health state

A mixed effects regression model was fit to the data to account for the repeated (non-independent) nature of the measurements for each participant over multiple timepoints. Random intercepts (but not random slopes) were included in the model specification. An autocorrelation structure (AR1) was used to account for single order correlations between timepoints. For each participant, the measures post baseline are likely dependent upon the preceding timepoint, while also allowing for the magnitude of the correlation to decline over time. A Tukey method was used to adjust the confidence intervals and *p* values for multiple comparisons. The means and differences presented in the table are model estimated means and differences, and not the raw observed means and differences for each timepoint. Bold *p* values indicate statistically significant changes from baseline

### Regression analysis by compliance status

An ad hoc exploratory analysis was performed on the pooled population with outcomes evaluated by compliance status (complete vs partial compliance).

Although there were limited numbers of Black and Hispanic participants, we were interested to see if there was any difference in compliance compared with the White/non-Hispanic participants. Participants who identified as Black or Hispanic (*n*=5, 21.7%) were marginally more fully compliant than the participants who identified as White and non-Hispanic (*n*=18, 78.2%) (60.0 vs 50.0%) at the 60-day follow-up.

By regression analysis, only the fully compliant group demonstrated statistically significant improvement in arm girth at 60 days (change –1.7 cm, *p*=0.050). More participants who were fully compliant experienced decreased arm girth than the partially compliant participants (91.7% [11/12] vs 40.0% [4/10]).

Both the partially compliant and fully compliant groups demonstrated statistically significant reductions (*p*≤0.001) from baseline in weight and BMI at both the 30- and 60-day follow-ups. There were no significant differences between compliance groups.

Improvements in the overall score for the LYMQOL-ARM questionnaire at 60-days were observed in 70% (7/10) of partially compliant and 50% (6/12) of completely compliant participants, as well as in the 1 noncompliant participant. By regression analysis, there were no significant differences within or between groups for the overall score at either time point. Only the functional domain score demonstrated a significant improvement over baseline for the fully compliant group at 60 days (change –0.38, *p*=0.030).

LSIDS-A overall scores were improved at 60-days for 77.8% (7/9) of the partially compliant participants (1 participant did not complete all questionnaire items) and 91.7% (11/12) of the completely compliant participants. By regression analysis, the change from baseline to 60-day follow-up for the fully compliant group was statistically significant for the soft tissue sensation (–1.6, *p*=0.037), neurological sensation (–1.8, *p*=0.013), and overall domains (–1.0, *p*=0.021) of the LSIDS-A. Changes from baseline for the partially compliant group did not reach statistical significance at either time point for any domain score.

For the SF-36 questionnaire, there were no statistically significant differences in any domains within or between compliance groups for either follow-up time point by regression analysis.

### Adverse events

During the study period, 7 device-related adverse events occurred in 5 participants. Events in the test group were 1 case each of suspected cellulitis, tenderness, and maculopapular rash. Events in the control group were 1 case each of clicking in thumb joint, bilateral buttock pain, worsening lymphedema, and exacerbation of arm pain. None of the events were serious and all were mild to moderate in severity.

## Discussion

Our study findings indicate that the text reminders did not improve treatment compliance because the BCRL patients were already highly treatment compliant. Overall, there were significant improvements in mean weight and BMI at the 30-day visit and in arm girth at the 60-day visit. There were significant improvements in several lymphedema-specific QOL and symptom severity measures and 30- and/or 60-day follow-ups and the SF-36 pain domain score was significantly improved at both the 30- and 60-day follow-ups.

Because we did not find any differences in compliance by the randomized study groups for the primary endpoint, the data were pooled and outcomes evaluated by compliance status (complete vs partial compliance). There were no differences between compliance groups for weight, BMI, and SF-36 scores, and LYMQOL-ARM scores except the functional domain. Compared to the partially compliant group, the fully compliant group experienced significantly greater improvements in arm girth, LYMQOL-ARM functional domain score, and in the LSIDS-A overall, soft-tissue sensation, and neurological sensation scores. Although even partial compliance is beneficial, these findings support the effort to encourage full treatment compliance among BCRL patients in order obtain the optimal benefits the therapy offers.

The strengths of this study include the randomized assignment and comparison of participants who received or did not receive text reminders for treatment compliance. Additionally, we measured compliance through data automatically received from the PCD device, mitigating recall issues of patient-reported compliance. We also included comparisons of validated patient-reported outcomes to evaluate changes in quality of life and symptomology related to treatment compliance.

One obvious limitation of the study is the small sample size. This study was designed as a small feasibility study to further inform additional studies in the future. Additionally, there is the potential for selection bias since all study participants were required to have the capability to receive text messages at their treatment location. This requirement effectively eliminated any patients who had limited internet or phone accessibility issues. Although we were interested to see if there were any differences in outcomes across racial and ethnic variables, our population was predominantly White, making it difficult to meaningfully assess for the presence of health disparities among those who met eligibility criteria. That said, we did see a trend toward higher rates of full compliance among Black study participants compared with White participants. Larger studies with more diverse populations may help to delineate if any equity issues exist with access to and compliance with PCD therapy.

Finally, in hindsight, the selection of the BCRL population was not the most effective group on which to test this technology since the population is already highly compliant without receiving reminders. Future studies in other populations of patients with lymphedema may demonstrate different results.

## Conclusion

Automated text reminders did not improve compliance in patients with BCRL as compliance rates were already high in this patient population across racial and ethnic variables. Improvements in weight, BMI, arm girth, disease-specific quality of life, and symptom severity measures were observed regardless of the treatment assignment. Full compliance resulted in greater functional and QOL benefits.
